# Orally Administered *Salacia reticulata* Extract Reduces H1N1 Influenza Clinical Symptoms in Murine Lung Tissues Putatively Due to Enhanced Natural Killer Cell Activity

**DOI:** 10.3389/fimmu.2016.00115

**Published:** 2016-03-31

**Authors:** Gustavo A. Romero-Pérez, Masayo Egashira, Yuri Harada, Takeshi Tsuruta, Yuriko Oda, Fumitaka Ueda, Takamitsu Tsukahara, Yasuhiro Tsukamoto, Ryo Inoue

**Affiliations:** ^1^Kyoto Institute of Nutrition and Pathology, Kyoto, Japan; ^2^Laboratory of Animal Science, Department of Agricultural and Life Sciences, Kyoto Prefectural University, Kyoto, Japan; ^3^Life Science Research Laboratories, Research and Development Management Headquarters, Fujifilm Corporation, Kanagawa, Japan; ^4^Laboratory of Animal Hygiene, Department of Agricultural and Life Sciences, Kyoto Prefectural University, Kyoto, Japan

**Keywords:** *Salacia reticulata* extract, H1N1 influenza virus, *Lactobacillus casei* JCM1134, antibiotics, gut microbiota, natural killer cells, splenocytes, pulmonary cells

## Abstract

Influenza is a major cause of respiratory tract infection. Although most cases do not require further hospitalization, influenza periodically causes epidemics in humans that can potentially infect and kill millions of people. To countermeasure this threat, new vaccines need to be developed annually to match emerging influenza viral strains with increased resistance to existing vaccines. Thus, there is a need for finding and developing new anti-influenza viral agents as alternatives to current treatments. Here, we tested the antiviral effects of an extract from the stems and roots of *Salacia reticulata* (SSRE), a plant rich in phytochemicals, such as salacinol, kotalanol, and catechins, on H1N1 influenza virus-infected mice. Following oral administration of 0.6 mg/day of SSRE, the incidence of coughing decreased in 80% of mice, and only one case of severe pulmonary inflammation was detected. Moreover, when compared with mice given *Lactobacillus casei* JCM1134, a strain previously shown to help increase *in vitro* natural killer (NK) cell activity, SSRE-administered mice showed greater and equal NK cell activity in splenocytes and pulmonary cells, respectively, at high effector cell:target cell ratios. Next, to test whether or not SSRE would exert protective effects against influenza in the absence of gut microbiota, mice were given antibiotics before being inoculated influenza virus and subsequently administered SSRE. SSRE administration induced an increase in NK cell activity in splenocytes and pulmonary cells at levels similar to those detected in mice not treated with antibiotics. Based on our results, it can be concluded that phytochemicals in the SSRE exerted protective effects against influenza infection putatively *via* modulation of the immune response, including enhancement of NK cell activity, although some protective effects were not necessarily through modulation of gut microbiota. Further investigation is necessary to elucidate the molecular mechanisms underlying the protective effects of SSRE against influenza infection.

## Introduction

Influenza is a leading cause of respiratory tract infection (1). Influenza viruses are categorized as A, B, and C (2), and influenza A virus can be further subtyped as H3N2, H2N2, and H1N1. Every year, approximately 10% of the world’s population is infected with influenza (3) that results in 250,000–500,000 deaths (4), but in most cases, the infection only lasts for 1–2 weeks without the need for hospitalization (4). Nonetheless, influenza viruses periodically cause epidemics in humans, such as the 2009 H1N1 pandemic in Mexico (5), which can potentially infect and kill millions of people. Thus, medication with antiviral agents that trigger an immune response and inhibit viral replication in infected patients is required to prevent further viral spread and higher mortality rates among the population.

Following an influenza viral infection, natural killer (NK) cells are reportedly activated by proinflammatory cytokines, such as interleukin-12 (6) and/or type I interferons (7), and cytotoxicity of NK cells is stimulated by interferon-gamma (IFN-γ) (8, 9). Since toxicity receptors NKp46 and NKp44 on human NK cells readily identify hemagglutinin and neuraminidase on the surface of influenza virus, NK cells alone have the potential of destroying infected cells (9). Nonetheless, impaired cytotoxicity and depletion of NK cells that lead to higher morbidity and mortality rates are often observed in influenza virus-infected subjects for reasons not yet fully understood (10). Hence, presently influenza viral infections are most efficiently prevented and controlled by vaccines that are mainly designed to mobilize a strain-specific antibody response to viral surface hemagglutinin or neuraminidase (2). Nevertheless, every year, new vaccines need to be developed to match emerging influenza viral strains with increased resistance to existing vaccines (11, 12). This challenge highlights the need for finding and developing new antiviral agents as alternatives to those currently available.

Although the human gut microflora is remarkably stable under dietary changes (13) and its composition is highly individualized (14), administration of lactic acid bacterial (LAB) strains, such as those found in fermented food products (15–17), has been shown to protect experimental models against influenza viral infections by enhancing the immune response (16–20). For example, continual consumption of a milk-based drink containing a *Lactobacillus brevis* strain was reported to lower the incidence of influenza in unvaccinated schoolchildren in Japan (15). These findings emphasize the potential of probiotics as complement to conventional vaccine-based approaches for treatment of influenza infection (21). Nonetheless, inconsistency in the survival rate of strains (22, 23) and lack of consensus on the effective doses (24, 25) are issues needed to be addressed before LAB can be considered reliable as therapeutic agents against viral infections.

A new generation of antiviral extracts from biological sources has shown promising effects against influenza virus. For example, Ladania067 from the leaves of black currant (*Ribes nigrum folium*) showed potent antiviral activity against influenza A/Regensburg/D6/09 (H1N1) (26), although efficacy observed 24 h after intranasal application *in vivo* was not higher than 85%. Likewise, a crude extract from *Crytoporus volvatus*, a polypore fungus, showed strong activity against H1N1/09 influenza viral infection in mice. Nonetheless, while *C. volvatus* extract indeed reduced virus loads in lungs and protected from a lethal viral dose, it failed to substantially decrease the virus titer (27). Although *Salacia* species have long been used as therapeutic agents in traditional medicine in Asia for treating disorders, such as diabetes, cancer, and immunosuppression (28, 29), the properties of *Salacia* species against viral infections have not been well characterized. Recently, in our premises, we observed that an extract from the bark and roots of *Salacia reticulata* induced an increase in activity of immune-related genes, including Cd26, IgG2a, TNF-α, and Ccl5 in intestinal epithelial cells (IEC) of Sprague-Dawley rats (30). Cd26 and IgG2a are involved in cell-mediated immunity (31) and the humoral immune response (32), and TNF-α and Ccl5 in the antiviral defense response (33), respectively, upon a challenge with influenza virus. Thus, it is likely that extracts from *S. reticulata* may induce a similar increase in immune cell activity after influenza virus infection.

In the present study, we first evaluated the effect of administration of an *S. reticulata* stem and root extract (SSRE) on lung damage caused by influenza viral infection. Next, we measured NK cell activity in spleen and lungs of influenza virus-infected mice to further assay the protective effect of administration of SSRE against influenza viral infection, and compared this effect with that exerted by a commercially available LAB strain. We also investigated the effect of SSRE on NK cell activity in spleen and lung cells of mice given a powerful antibiotic cocktail and inoculated with H1N1 influenza virus.

## Materials and Methods

### Animals

Six-week-old ddY mice were purchased from Japan SLC (Shizuoka, Japan) and bred in a pathogen-free animal facility. Mice had *ad libitum* access to regular rodent chow (Lab MR stock; Nihon Nosan Kogyo, Tokyo, Japan) and water. Prior to experiments, all mice were allowed to acclimatize for 7 days. The experimental animals were handled in accordance with the guidelines for animal studies issued by the Experimental Animal Committee of Kyoto Prefectural University (Approval No. KPU270406).

### Bacterial and Viral Strains

*Lactobacillus casei* JCM1134 was purchased from Japan Collection of Microorganisms (Tsukuba, Japan). *L. casei* was grown under anaerobic conditions at 37°C overnight in 10-mL Hungate tubes, with de Man, Rogosa, and Sharpe (MRS) broth as medium.

A murine influenza virus, A/PR/8/34/2009 (H1N1), was used for this study. Madin–Darby canine kidney cells were infected with the virus and after several passages, viral solutions were prepared and titrated as the tissue-culture-infective dose (TCID_50_), according to a standard procedure (34, 35).

### Preparation of the Hot Water Extract of *S. reticulata*

The stems and roots of *S. reticulata* grown in and imported from Sri Lanka were dehydrated and pulverized. The powdered material was boiled in water at 90°C for an hour. The concoction was then filtered to remove any solid material, and the resulting filtrate was spray-dried (ADL-310, Yamato Science, Tokyo, Japan). The SSRE was stored at 4°C until further use.

### Experimental Design

#### Experiment 1

Twelve mice were equally divided and allocated to two experimental groups. Mice were orally administrated treatments as follows.

(1) Control group treatment: 500 μL of phosphate-buffered saline (PBS; 137 mM NaCl, 2.7 mM KCl, 10 mM Na_2_HPO_4_, and 1.8 mM KH_2_PO_4_; pH 7.4).
(2) SSRE group treatment: 500 μL of SSRE solution (1.2 mg of SSRE/mL of PBS).

#### Experiment 2

Eighteen mice were equally divided and allocated to three experimental groups. Mice were orally administrated treatments as follows.

(1) Control group treatment,
(2) SSRE group treatment,
(3) Probiotic group treatment: 10^8^ cfu of JCM1134 in 500 μL of PBS.

#### Experiment 3

Eighteen mice were equally divided and allocated to three experimental groups. Mice were orally administrated treatments as follows.

(1) Antibiotic–control group (baseline group) treatment: antibiotics in drinking water [1 g/L of ampicillin (Nacalai Tesque, Kyoto, Japan), 500 mg/L of vancomycin (Wako Pure Chemicals, Osaka, Japan), 1 g/L neomycin sulfate (Sigma-Aldrich, Tokyo, Japan), and 1 g/L of metronidazole (Nacalai Tesque, Kyoto, Japan)] + control group treatment.
(2) Antibiotic–SSRE group treatment: antibiotics + SSRE.
(3) Normal microbiota group treatment: control group treatment.

In Experiment 3, administration of antibiotics started 3 days prior to administration of SSRE. Treatments in all experiments were administered once a day for 2 weeks. The humane endpoint was established at a body weight loss >20%, at which mice would be killed, as previously suggested (36).

### Experimental Influenza Viral Infection to Mice

After completion of the treatment period, mice from all experiments were intranasally inoculated with 10^5^ TCID_50_ of A/PR/8/34/2009 (H1N1) virus. Five days post-inoculation, mice were killed with an overdose of sodium pentobarbital (Schering-Plough, K.K. Osaka, Japan). Except for tissues from Experiment 1, which were immersed immediately after removal in 10% neutral-buffered formalin and used for histopathology analysis, all murine lungs and spleen tissues of mice were collected and immediately used for further analyses.

### Histopathology

Lung tissues of mice from Experiment 1 were used for this experiment. Lung tissues were embedded in paraffin, and 5-μm sections were excised from bilateral posterior lobes and stained with hematoxylin and eosin (H&E). All samples were randomly numbered and examined by an experienced pathologist blinded to the study conditions. The pathologist used an Olympus microscope (Olympus Optical Co., Ltd., Tokyo, Japan) with a 100× magnification lens and scanned the entire surface area of each lung section.

### Isolation of Splenocytes and Pulmonary Cells

Lung and spleen tissues were sectioned into small pieces and immersed in ice-cold Hanks buffered solution (HBSS, Nacalai Tesque, Kyoto, Japan). The pieces were gently pressed against sterile 70-μm cell strainers (BD Biosciences Japan, Tokyo, Japan) to obtain single-cell suspensions, which were centrifuged at 500 × *g* for 4 min at 4°C. The cell supernatant was retrieved, resuspended in ACK lysis buffer (0.5 mol/L of NH_4_Cl, 10 mmol/L of KHCO_3_, and 0.1 nmol/L of Na_2_EDTA; pH 7.2), and incubated at room temperature for 5 min to remove erythrocytes. The remaining cells were washed twice with HBSS and resuspended in 1 mL of culture medium [RPMI medium (Nacalai Tesque) containing 10% fetal bovine serum (FBS), penicillin (100 U/mL, Sigma-Aldrich Japan, Tokyo, Japan), and streptomycin (100 μg/mL; Sigma-Aldrich, Japan)]. Splenocytes and pulmonary cells were stained with trypan blue and counted with a TC10 automated cell counter (Bio-Rad, Richmond, CA, USA).

### NK Activity of Splenocytes and Pulmonary Cells

A murine T cell lymphoma cell line, YAC-1, was obtained from RIKEN Bio-Resource Center (Ibaraki, Japan). YAC-1 cells were cultured in 75-cm^2^ culture flasks containing 10 mL of culture media and incubated in a humidified chamber (37°C, 5% CO_2_). After 3-day incubation, YAC-1 cells were stained for 15 min at 37°C with 10-μM Dioc18 [3,3′-dioctadecyloxacarbocyanine perchlorate (Life Technologies Japan, Tokyo, Japan)] to cause green fluorescence of cell membranes. YAC-1 cells were then washed twice with 1-mL HBSS and seeded in 100 μL of culture media in 96-well culture plates (Orange Scientific, Tokyo, Japan) in a concentration of 1.0 × 10^4^ cells (for coculture with splenocytes) or 5.0 × 10^3^ cells (for coculture with pulmonary cells) per well.

**Figure 1:**
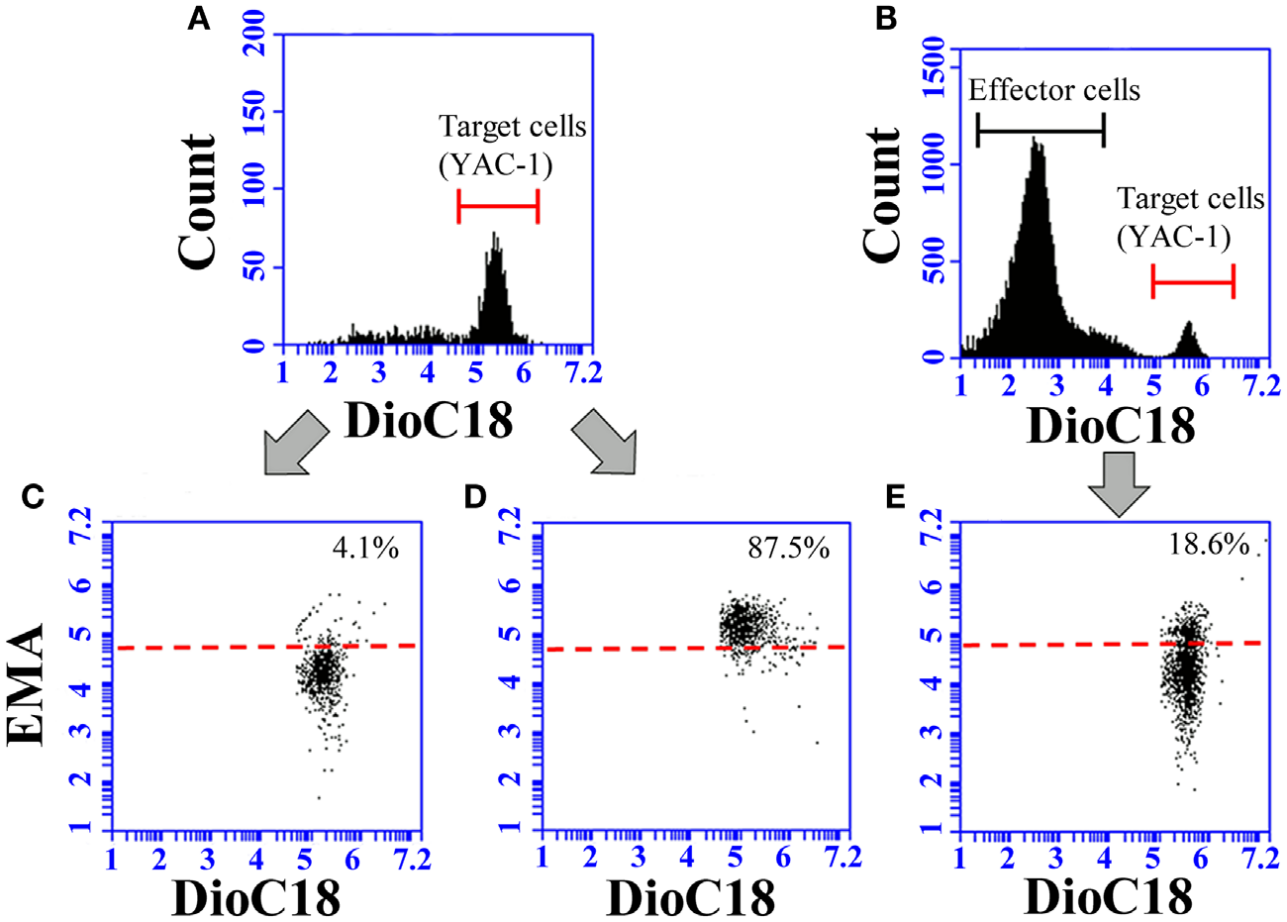
**Representative images of flow-cytometric analysis for the determination of NK activity**. To discriminate the target cells, live gate (red line) was set in the FL1 histogram on the green fluorescent **(A,B)**. The target cells were further analyzed with a dot plot of FL1 and FL3 **(C–E)**. Spontaneous lysis of YAC-1 cells during the incubation was determined for DioC18-labeled YAC-1 cells incubated in the absence of effector cells **(C)**. The gate for the discrimination of live and dead target cells (red dotted line) was set in accordance with the red fluorescence of DioC18-labled YAC-1 cells treated with 3% saponin **(D)**. The target cells lysed by effector cells appear in the gate for dead cells **(E)**.

Natural killer cell activity was assessed by a flow-cytometric method, as previously described (37, 38). For the NK activity assays, YAC-1 cells were used as target cells, and splenocytes and pulmonary cells as effector cells. One hundred microliters each of splenocyte and pulmonary cell suspensions were added to each well containing DioC18-labeled YAC-1 cells to achieve 25:1 and 10:1, and 10:1 and 5:1 effector cell:target cell (E:T) ratios, respectively. After incubation for 4 h, 10 μL of ethidium monoazide bromide (EMA, 0.5 ng/mL, Sigma-Aldrich, Japan) solution was added to each well for nuclear staining of dead cells. The ethidium was bound to nuclei by photo affinity using a 26-W fluorescent light (5 cm distance) for 10 min at room temperature. After washing twice with PBS containing 0.5% BSA (Nacalai Tesque), the cells were fixed with 2% PFA overnight. Each sample was incubated in duplicate and analyzed using an Accuri C6 flow cytometer (Beckton Dickinson). During the data acquisition, a live gate was set in the FL1 histogram on the green fluorescent target cells to discriminate effector and target cells (Figures 1A,B). At least 2,000 target cells per sample were collected. The gated target cells were further analyzed with a dot plot of FL1 (green fluorescence) versus FL3 (red fluorescence) to calculate the percentage of EMA-positive dead cells.

The percentage of spontaneously lysed target cells was determined for DioC18-labeled YAC-1 cells incubated for 4 h in the absence of effector cells (Figure 1C), and the value was subtracted from the data of each sample. Spontaneous lysis of target cells ranged from 3 to 10% depending on the experiment. DioC18-labeled YAC-1 cells treated with 3% saponin were also stained with EMA and further analyzed to set the gate for discrimination of live and dead cells (Figure 1D). A representative dot plot image of the target cells after incubation with effector cells is shown in Figure 1E.

### Statistical Analysis

Data of NK activity were analyzed by one-way ANOVA followed by Tukey HSD *post hoc* test to determine significant differences among the treatment groups. In all statistical analyses, differences were considered significant if *P* values were <0.05. All data were analyzed using freely available R software version 3.1.2 (https://www.r-project.org/).

## Results

### Effect of *Salacia* Extract

Five days post-infection, mice infected with influenza virus showed noticeable clinical symptoms, including sneezing and coughing. Nonetheless, upon oral administration of 0.6 mg/day of SSRE, 80% of mice showed a decrease in the incidence of coughing (Table 1).

**Table 1:** **Effect of *Salacia* stem and root extract on influenza virus-infected mice**.

Treatment	Concentration (mg/day)	Infected mice	Viral dose (TCID_50_)	Number of mice				
				Symptoms		Pulmonary inflammation		
				Sneezing	Coughing	Normal	Moderate	Severe
Control	0	5	10^5^	1	5	0	0	5
SSRE	0.6	5	10^5^	1	1	0	4	1

*SSRE, *S. reticulata* stem and root extract*.

### Histopathology

**Figure 2:**
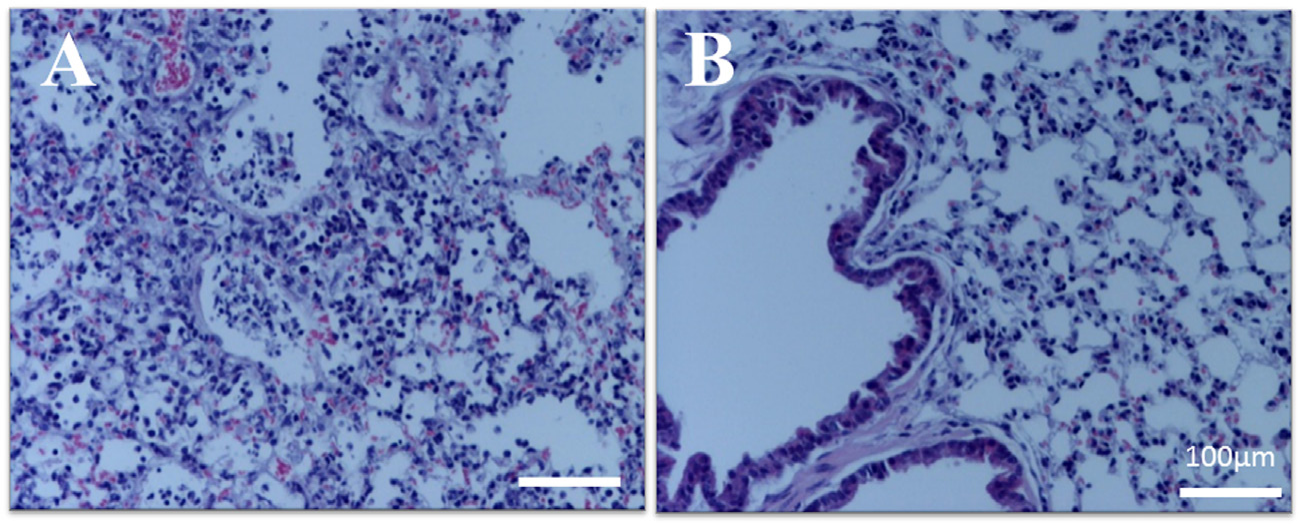
**Pulmonary histopathology of influenza virus-infected mice**. Influenza virus-infected mice were administered an *S. reticulata* stem and root extract (SSRE), and their lung tissues were examined histopathologically 5 days post-viral infection. **(A)** Control group: pulmonary tissue sections from control mice show acute influenza pneumonia. In addition, infiltration of inflammatory cells, such as neutrophils and macrophages, can be observed in the alveoli, along with associated edema and cell debris. **(B)** SSRE group: only slight inflammation was observed in the pulmonary tissue sections of SSRE-administered mice.

Histopathologically, pulmonary tissues of control mice showed typical acute influenza-associated pneumonia, as neutrophilic and lymphocyte infiltration was prominent in the alveoli and peri-bronchi (Figure 2A). Edema and cell debris were also noted. In contrast, pulmonary inflammation was clearly inhibited in mice in the SSRE group (Figure 2B), and the influenza cases in this group decreased from five severe to one severe and four moderate (Table 1).

### NK Cell Activity Assays

#### Comparison between the Effects of *Salacia* Extract and Lactic Acid Bacteria Preparation

**Figure 3:**
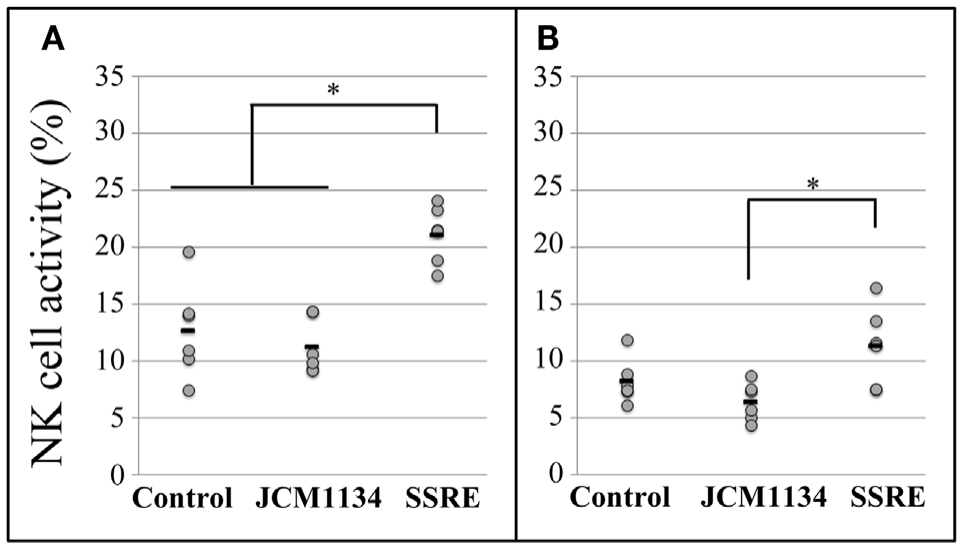
**Natural killer cell activity in splenocytes of influenza-infected mice administered either *L. casei* JCM1134 or *S. reticulata* stem and root extract**. **(A)** NK cell activity in splenocytes of mice at an effector cell:target cell (E:T) ratio of 25:1. **(B)** NK cell activity in splenocytes of mice at an E:T ratio of 10:1. Control: mice given 500 μL of phosphate-buffered saline (PBS). JCM1134: mice administered 10^8^ cfu of *L. casei* JCM1134 in 500 μL of PBS. SSRE: mice administered 500 μL of *S. reticulata* stem and root extract in PBS (1.2 mg/mL of PBS). Each horizontal bar represents the mean value for six mice. *P < 0.05.

At the E:T ratio of 25:1, NK cell activity in splenocytes of SSRE-administered mice was significantly higher than that observed in splenocytes of mice given JCM1134 or in the control group (Figure 3A). At the E:T ratio of 10:1, splenocytes of SSRE-administered mice had higher NK cell activity than did those of mice administered JCM1134, although this difference was not significantly higher than the NK cell activity detected in splenocytes of control mice (Figure 3B).

**Figure 4:**
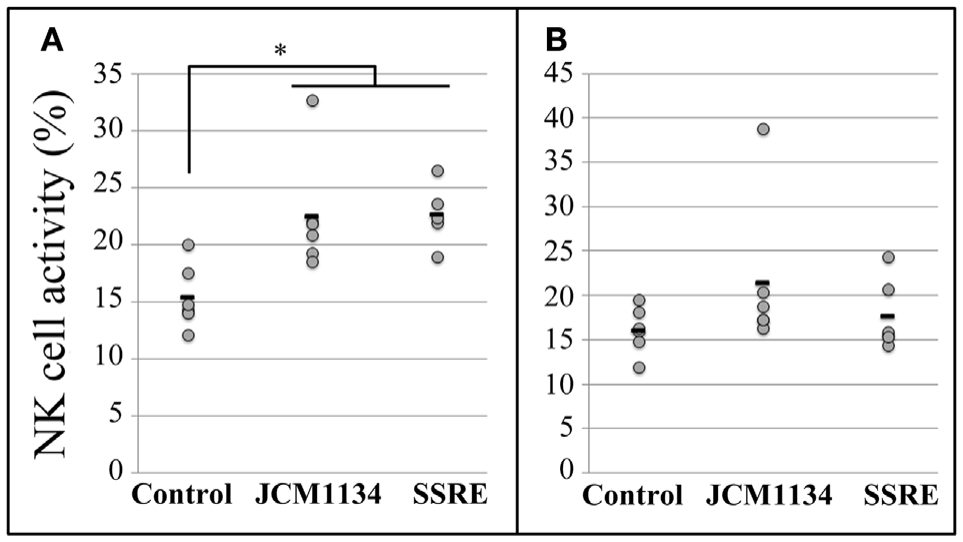
**Natural killer cell activity in pulmonary cells of influenza-infected mice administered either *L. casei* JCM1134 or *S. reticulata* stem and root extract**. **(A)** NK cell activity in pulmonary cells of mice at an effector cell:target cell (E:T) ratio of 10:1. **(B)** NK cell activity in pulmonary cells of mice at an E:T ratio of 5:1. Control: mice given 500 μL of phosphate-buffered saline (PBS). JCM1134: mice administered 10^8^ cfu of *L. casei* JCM1134 in 500 μL of PBS. SSRE: mice administered 500 μL of *S. reticulata* stem and root extract in PBS (1.2 mg/mL of PBS). Each horizontal bar represents the mean value for six mice. *P < 0.05.

Regarding the NK cell activity in pulmonary cells, at the E:T ratio of 10:1, it increased in mice administered both SSRE and JCM1134 when compared with the NK cell activity in pulmonary cells of mice in the control group (Figure 4A). However, at the E:T ratio of 5:1, the NK cell activity was comparable across all treatment groups (Figure 4B).

### Effect of *Salacia* Extract upon Administration of an Antibiotic Cocktail

The body weight of mice drastically decreased after antibiotic administration. As a result, most tissues, including spleen, were substantially small and thus, collecting a sufficient number of cells from them for the analyses proved difficult. Hence, to ensure reliability and based on the results from Experiment 2, it was decided to use splenocytes and pulmonary cells only at E:T ratios of 25:1 and 10:1, respectively.

**Figure 5:**
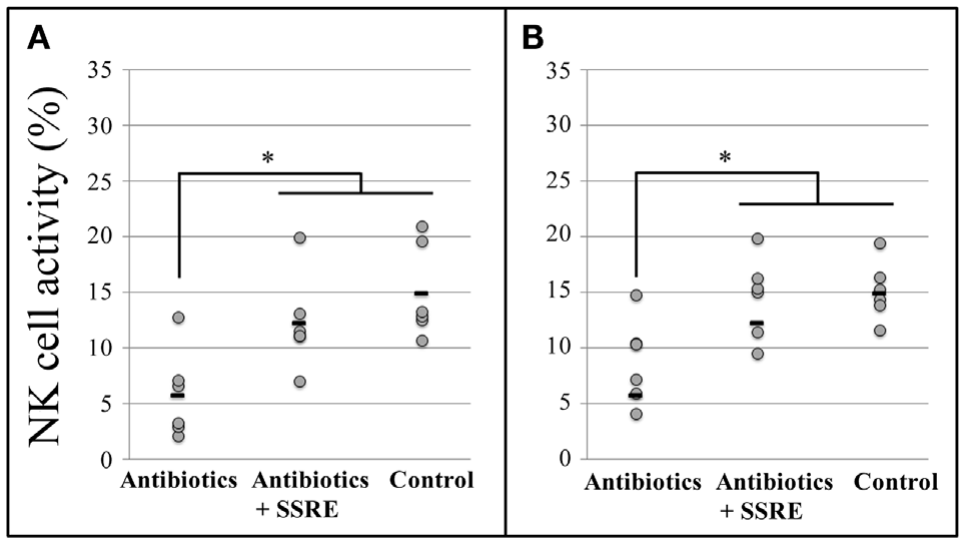
**Natural killer cell activity in splenocytes and pulmonary cells of influenza-infected mice administered *S. reticulata* stem and root extract after given an antibiotic cocktail**. Antibiotic administration drastically decreased the body weight, and hence, the size of tissues of mice also decreased. As a result, the number of splenocytes and pulmonary cells for the analyses was insufficient. Thus, splenocytes **(A)** and pulmonary cells **(B)** were used only at effector cell:target cell (E:T) ratios of 25:1 and 10:1, respectively. Antibiotics: mice given an antibiotic cocktail in drinking water consisting of 1 g/L of ampicillin, 500 mg/L of vancomycin, 1 g/L neomycin sulfate, and 1 g/L of metronidazole + 500 μL of phosphate-buffered saline (PBS). Antibiotic–SSRE: mice administered the antibiotic cocktail + 500 μL of *S. reticulata* stem and root extract in PBS (1.2 mg/mL of PBS). Control: mice with intact microbiota given 500 μL of phosphate-buffered saline (PBS). Each horizontal bar represents the mean value for six mice. *P < 0.05.

At the 25:1 E:T ratio, SSRE administration induced an increase in NK cell activity in splenocytes of antibiotic-administered mice that reached a level similar to that detected in splenocytes of mice not treated with antibiotics (Figure 5A). Likewise, at the 10:1 E:T ratio, NK cell activity in pulmonary cells of antibiotic-treated mice was increased by SSRE administration to a level similar to that detected in splenocytes of mice not given the antibiotic cocktail (Figure 5B).

## Discussion

*Salacia* species were previously found to have potential immunomodulatory properties for treating influenza *in vitro* (39, 40). At these premises, we also observed that administration of a bark and root extract from *S. reticulata* triggered an ­antiviral immune response in rat ileum involving Cd26 and IgG2a (30). Therefore, we wanted to test whether SSRE would trigger similar immune responses in H1N1 influenza-inoculated mice.

Excessive production of immune cells, such as neutrophils and lymphocytes, during influenza infection often leads to severe lung inflammation (41). In contrast, our results showed that upon SSRE administration, pulmonary inflammation of lung tissues markedly decreased (Figure 2B) and cough scores improved (Table 1) in influenza-inoculated mice. Similar effects were previously reported in influenza virus-infected mice that administered a compound prepared with extracts from plant roots and rice grains (42). It is very likely that SSRE was effective in reducing production of immune cells by inhibiting influenza viral replication. For example, in previous work, it was reported that *Salvia chinenesis* inhibited the binding of viral double stranded RNA and non-structural protein 1 (39) expressed in nuclei of H5N1 virus-infected cells (43). Non-structural ­protein 1 is essential for the virus replication cycle (44), blocks gene expression (45), and antagonizes innate immune response (46) initiated in host cells.

In the present study, we focused on NK cell activity to further evaluate the protective effect of SSRE against H1N1 influenza virus-infected mice. NK cells are the first line of defense against influenza infection (7). However, if NK cell activity is not regulated, healthy cells may also be damaged by NK cell cytotoxicity (47). While we observed that SSRE administration substantially enhanced NK cell activity, histological observation confirmed that there was no excessive infiltration of cells in lung tissues of SSRE-administered mice. Hence, we believe that although SSRE administration induced an increase in NK cell activity, it also activated regulatory processes that maintained the number of NK cells produced under control. A preliminary experiment conducted in our laboratory provided further evidence for this theory, as it suggested that SSRE administration did not alter NK cell activity in splenocyte and pulmonary cells of healthy mice, that is, mice not infected with influenza virus (data not shown). In other words, this result seems to indicate an absence of overstimulation by SSRE of NK cell activity under healthy conditions. Our results also showed that at high E:T ratios, NK cell activity was enhanced in splenocytes and pulmonary cells of SSRE-administered mice, but at lower E:T ratios, NK cell activity was not different across treatment groups. During viral infections, cell-to-cell contact between NK cells and infected cells downregulates receptors on NK cell surface, such as NKG2D and NKp30, which inhibits NK cell activity *in vivo* (48). Thus, high effector/target ratios at primary infection sites are required for an effective immune protection (49), which is consistent with our results.

In Experiments 1 and 2, it is very probable that gut microbiota of mice played a role in the effects observed after administration of SSRE. Evidence suggests that during viral infection, gut microbiota helps reduce host susceptibility by exerting *colonization resistance* (50) to prevent adhesion of pathogens and maintain immune homeostasis. Indeed, gut microbiota has been suggested to upregulate the expression of NK cell surface ligands on IEC when microbiota imbalance and intestinal inflammation are detected (51). Influenza virus infection, however, can severely alter the composition of gut microbiota and disrupt gut homeostasis. For example, previous studies showed that influenza virus infection increased exposure of galactose and mannose residues on IEC surfaces and hence overexpression of glycoreceptors, which increased adhesion of pathogens and caused an elevated production of proinflammatory cytokines (52).

Certain LAB strains with probiotic activity also show antiviral properties. Moreover, LAB supplementation to influenza-infected models has been reported to help protect against influenza infections. For example, LAB preparations were demonstrated to modulate immune responses in influenza virus-infected mice (16–19), which resulted in lower virus titer (16) and improved clinical symptoms (20). Therefore, to estimate the immunomodulatory properties of SSRE, in Experiment 2, we tested SSRE against JCM1134, a bacterial strain previously used elsewhere as adjuvant to help increase *in vitro* NK cell activity (53). Interestingly, our results showed that NK cell activity induced by SSRE was similar to or greater than that induced by JCM1134, especially in spleen, which suggests that SSRE could be used as an alternative or complement to LAB supplementation to treat influenza infections.

Although detailed investigation of the molecular mechanisms of action of individual active ingredients in SSRE was beyond the scope of the present work, two routes can be theorized. *S. reticulata* is rich in phytochemicals with immunomodulatory properties, such as polyphenols salacinol, kotalanol (54, 55), epigallocatechin, and epicatechin (56). Thus, one route may have been an indirect effect by salacinol and kotalanol. Salacinol and kotalanol likely inhibited disaccharidases, including α-glucosidase, in the gut of mice (54, 55, 57), which altered the composition of gut microbiota, such as the ratio between firmicutes and bacteroidetes (30). This shift in microbial composition resulted in an increased release of activated cytokines, such as NKT cell-induced IFN-γ in IEC (9).

An alternative route may have been a more direct effect of phytochemicals in SSRE, as they were likely absorbed unaltered. Indeed, phytochemicals, such as catechins, may have stimulated NK cell immune surveillance levels (58) which, upon detection of viral particles, inhibited further replication of influenza virus (59). To test this alternative route, in Experiment 3, mice were administered SSRE after being given a powerful antibiotic cocktail and inoculated with influenza virus. We confirmed by fecal smear assays that the antibiotic cocktail removed most gut microbiota, because at the start and end of SSRE/PBS administration, only a negligible number of bacteria were found in feces of antibiotic-administered mice (data not shown). Moreover, as expected, the sole administration of antibiotics drastically inhibited NK activity in mice (Figure 5), the result of which was chosen as baseline to test the effect of SSRE administration. The significant reduction in NK activity detected in microbiota-deprived mice was likely due to the microbiota composition was severely altered by the antibiotic administration (60), which in turn caused downregulation of gene expression and disruption of the immune response (61). Antibiotics, such as azithromycin (62) and neomycin (63), reportedly suppress NK cell cytotoxicity and vancomycin decreases IFN-γ levels (51). Thus, the fact that the administration of SSRE increased NK cell activity to normal levels in splenocytes and pulmonary cells of microbiota-deprived mice was somewhat unexpected (Figures 5A,B). These results strongly suggest that phytochemicals in SSRE, possibly catechins, stimulated without mediation of gut microbiota an increase in NK cell activity in spleen and lungs. Moreover, these results are in concordance with previous work reporting the effectivity of a mixed extract from three edible plants, namely, *Angelica radix*, *Cnidium rhizome*, and *Paeonia radix*, in helping restore the proliferative response, cytokine production, and NK cell activity in splenocytes of aged mice with impaired immune response (64). Further investigation of the antiviral activity of phytochemicals in *S. reticulata* is needed to determine the underlying molecular mechanisms of action against influenza infection.

In summary, in the present work, we assayed the antiviral properties of an extract from stems and roots of *S. reticulata*. We showed that SSRE administration markedly reduced clinical symptoms and immune cells infiltration in lung tissues of influenza virus-infected mice. In addition, SSRE administration induced an increase in NK cell activity in splenocytes and pulmonary cells of mice equal to or greater than that observed in JCM1134-administered mice. The enhancement of NK cell activity by SSRE may contribute at least to some extent to the attenuation of lung damage observed in Experiment 1. Furthermore, when SSRE was given to mice deprived of gut microbiota by an antibiotic cocktail, NK cell activity was increased to a level similar to that observed in mice not given antibiotics. It can be reasonably concluded that at least some of the protective effects exerted by phytochemicals in SSRE against influenza infection were not necessarily through modulation of gut microbiota, but the underlying molecular mechanisms need to be further elucidated.

## Author Contributions

GR-P: wrote the paper, analyzed the data, helped complete the experiments, and prepared and edited figures and tables. ME, YH and TTsuruta: carried out the experiments. YO and FU: conceived the experiment and prepared the experimental material (*Salacia*). TTsukahara: conceived and co-designed the experiment. YT: conceived and co-designed the experiment and prepared influenza virus inoculant. RI: conceived and co-designed the experiment, supported during the experiments, co-wrote the paper, and supervised data analysis.

## Conflict of Interest Statement

The present work was conducted in the absence of any commercial or financial relationship that could be construed as a potential conflict of interest.
